# Change of self-rated physical health predicts mortality in aging individuals: results of a population-based cohort study

**DOI:** 10.1186/s13690-024-01363-9

**Published:** 2024-08-23

**Authors:** Anna Celine Reinwarth, Felix S. Wicke, Kamiar K. Rückert, Jörn M. Schattenberg, Oliver Tüscher, Philipp S. Wild, Thomas Münzel, Jochem König, Karl J. Lackner, Norbert Pfeiffer, Manfred E. Beutel

**Affiliations:** 1 Department of Psychosomatic Medicine and Psychotherapy, University Medical Center of the Johannes Gutenberg-University Mainz, Zahlbacher Straße 8, 55131 Mainz, Germany; 2 Metabolic Liver Research Program, University Medical Center of the Johannes Gutenberg-University Mainz, Mainz, Germany; 3 Department of Internal Medicine II, Saarland University Medical Center, Homburg and University of the Saarland, Saarbrücken, Germany; 4 Department of Psychiatry and Psychotherapy, University Medical Center of the Johannes Gutenberg-University Mainz, Mainz, Germany; 5 Institute of Molecular Biology (IMB), Mainz, Germany; 6 Leibniz Institute for Resilience Research (LIR), Mainz, Germany; 7 Preventive Cardiology and Preventive Medicine, Department of Cardiology, University Medical Center of the Johannes Gutenberg-University Mainz, Mainz, Germany; 8 Center for Thrombosis and Hemostasis, University Medical Center, Johannes Gutenberg-University Mainz, Mainz, Germany; 9 German Center for Cardiovascular Research (DZHK), Partner Site Rhine-Main, Mainz, Germany; 10 Department of Cardiology - Cardiology I, University Medical Center of the Johannes Gutenberg- University Mainz, Mainz, Germany; 11 Institute of Medical Biostatistics, Epidemiology and Informatics, University Medical Center of the Johannes Gutenberg-University Mainz, Mainz, Germany; 12 Institute of Clinical Chemistry and Laboratory Medicine, University Medical Center of the Johannes Gutenberg-University Mainz, Mainz, Germany; 13 Department of Ophthalmology, University Medical Center, Johannes Gutenberg University Mainz, Mainz, Germany

**Keywords:** Course of self-rated physical health, Predictors of mortality, Sex-specific, Aging population, Cohort study

## Abstract

**Background:**

Self-rated physical health (SRPH) is known as an important predictor of mortality. Previous studies mostly used baseline values of self-rated health to predict long-term mortality. The effect of change in self-rated physical health on mortality during the course of aging has rarely been researched. The present study aimed to determine SRPH over time in women and men of an aging population, assess whether and how change in SRPH affects mortality while adjusting for known determinants of mortality, and test effect modification by sex on the relation between course of SRPH and mortality.

**Methods:**

Data of *N* = 12,423 respondents of the 5-year follow-up of the Gutenberg Health Study (GHS) with participation at the baseline assessment were analysed. All-cause mortality from 5-year follow-up onwards was defined as the primary outcome. SRPH was assessed by a single item. Cox proportional hazards models with adjustment for age, sex, socio-economic status and physical diseases were fitted to assess the predictive power of baseline score and course of SRPH. Additionally, effect modification by sex was assessed.

**Results:**

During a median follow-up period of 7.3 years (quartiles 6.0-8.5 years), 618 (5%) participants died. Overall, 70.9% of the participants indicated good or very good SRPH at baseline (T1) and follow-up (T2), 6.9% rated their SRPH as not so good at T1 and T2, and 0.6% reported bad SRPH at T1 and T2. An improvement of SRPH was indicated by 9.6% and 12.0% indicated deterioration of their SRPH. Change in SRPH added substantial predictive information to the Cox proportional hazards models, when adjusting for relevant covariates. In men, deterioration and constantly bad SRPH were associated with the strongest increase in risk of mortality by 87%, resp. 228%. While improvements increased mortality risk in men (67%), women with an improved SRPH had a lower risk (57%).

**Conclusion:**

A sizeable subgroup of aging participants reported deterioration of SRPH over five years. The association between change of SRPH and mortality is modified by sex. Deterioration of SRPH predicts mortality over baseline-assessment even when adjusted for relevant covariates. SRPH should be assessed regularly as part of an older individual’s health evaluation. Deterioration, constantly bad and improved SRPH should be taken seriously as unfavorable prognostic indicators, the latter only in men.

**Table:** 

Taxt box 1. Contributions to the literature
- There is limited evidence on the association of change in self-rated physical health and mortality risk, especially in Germany. Furthermore, the role of sex on the association has been neglected.
- Change in self-rated physical health is associated with increased mortality in the long run among German adults.
- In men, an improvement of self-rated physical health should be taken seriously as unfavourable prognostic indicator.
- Self-rated physical health is an important additional health indicator beyond clinical assessment and should be checked in medical consultations routinely.

## Introduction

To date, self-rated health has been recognized as an important predictor of quality of life, adverse health outcomes (e.g. diabetes, cardiovascular diseases, functional decline, depression) and mortality in adulthood [1–9]. Hence, within in an aging population, self-rated health has become a commonly used indicator of successful aging [10, 11]. It describes individuals’ subjective assessment of their health including physical, mental, cultural and social aspects of health [12–15]. Self-rated health is mostly assessed by single item questions, e.g. “In general, how would you rate your health today?” with response options such as very good or good, moderate, poor or very poor [16]. Its validity has been proven extensively [17] and shown to be stable among different age-groups and sex [18, 19]. A multitude of socio-economic and individual factors influence self-rated health. Poor self-rated health was more often reported by older adults and women compared to younger age groups and men [15, 20, 21]. Further, low education and income [22], unemployment [23], multimorbidity [24], health behavior [25], as well as mental health burdens were associated with poor self-rated health [20, 26]. However, the influence of these factors on self-rated health varies among individuals of different age cohorts in Europe. While age showed the strongest effect within adults born before 1945, education and employment were found to be strongest determinants in the age cohort of 1945 to 1964 and smoking was negatively associated among the younger generations born after 1965 [25].

Poor self-rated health has been consistently linked to higher all-cause mortality [27–29] and cardiovascular as well as cancer mortality [27, 30]. Compared to excellent self-rated health, poor self-ratings of health were associated with a two-fold higher mortality risk [31]. When relevant clinical correlates of mortality (e.g., co-morbidity, depression, subclinical illness or functional status) were taken into account, the association of self-rated health and mortality remained, even though its predictivity decreased [1, 32]. Analyzing cross-classification between self-rated health and objective health status for potential risk stratification, Mutz and Lewis [2] showed that, individuals with excellent self-rated health and favourable health status had lower mortality risk compared to individuals with poor self-rated health and unfavourable health status. The relation between self-rated health and mortality varied among individuals of different populations. For instance, poor self-rated health is more predictive of mortality in men than in women [12, 33, 34]. Further, authors of a French cohort study comparing the predictive value of self-rated health for mortality between different socio-economic groups over a mean period of 17.2 years, found weaker predictive ability within higher occupation, income and education groups [35].

Self-rated health may capture health aspects relevant to mortality which are not covered by clinical assessment [15]. However, most studies have not distinguished between the dimensions of self-rated physical and mental health when predicting mortality. Hence, using data of the Gutenberg Health Study (GHS), a large, prospective community cohort study in mid-Germany, we have already analysed the predictive value of the two self-rated health dimensions separately and showed that only self-rated physical, but not mental health is predictive of mortality, after controlling for relevant covariates [34]. Also, results of the Rotterdam Study suggested, that self-rated mental health is not predictive of mortality when sociodemographic, major chronic physical diseases, functional status, and mental health indicators were taken into account [36].

Research mainly used baseline measures of self-rated health to predict long-term mortality and treated self-rated health as a time constant variable, even when multiple assessments were available [12, 37]. However, self-rated health can be expected to change over the course of aging. Using data from the European Social Survey, Bauknecht and Merkel [38] examined the development of self-rated health of older persons in 17 countries between 2002 and 2018. Overall, an improvement of self-rated health was found in older subjects from different cohorts. Furthermore, in a recent Swedish study, which investigated change in self-rated health among older adults over a 20-year study period, 42.6% reported stable self-rated health, 40.6% deterioration, and 16.8% indicated an improvement [39]. Sperlich and Tetzlaff [40] analyzed age- and sex-specific longitudinal changes in self-rated health in Germany over 20 years based on 29,251 women and 26,967 men in the German Socio-Economic Panel. The authors found increasing self-rated health over time among women aged 41–50 to 71–80 years, while men reported improvements at the ages 61–70 and 71–80 years. No improvements were shown in the youngest (31–40 years) and oldest age group (81–90 years) in both sexes. To date, a multitude of studies tried to explain these changes by taking further socio-economic characteristics into account. For instance, a cohort study from the US determined changes in self-rated health in a sample of older adults (50–84 years) between 1972 and 2018 and showed that self-rated health has steadily improved over the 46-year study period, especially among participants with 13 years of education or more [41]. Also, Lamidi [42] highlighted the role of marital status and education in changes in self-rated health. While a recent systematic review concluded that the predictive impact of self-rated health on mortality is persistent in the medium and long-term perspective [43], other studies noted that the long-term predictive value of self-rated health on mortality was poorer than the short-term prediction [29, 37, 44]. Thus, independent effects of change in self-rated health on the association of self-rated health and mortality risk might have been disregarded.

To date, only a few studies analysed the association of change in self-rated health and mortality risk. For instance, Vogelsang [45] analysed the relationship between self-rated health change and mortality in a nationally representative sample of U.S. oldest-old adults and found that, adults indicating a decline in self-rated health had the greatest mortality risk. Interestingly, adults reporting an improvement in their self-rated health died more often than those who indicated no change. The author suspected that improvements may have been associated with surviving and recovering from a major health event (e.g. diagnoses of cancer, stroke, lung disease) or with normalizing pre-existing conditions [46, 47]. Thus, it can be assumed that sources of change may have independent associations with mortality [48]. Further, these initially slightly confusing findings underscore the fact that self-rated health improvements imply worse prior health ratings [49], that may still increase the risk of mortality compared to an unchanged or good health status. Additionally, Vogelsang [45] found no statistically significant differences between participants with an improved and participants with an unchanged self-rated health in respect to number of chronic physical health conditions, limited mobility or activities of daily living suggesting that improvements are more influenced by other factors. Especially with increasing age, individuals tend to change their subjective health norms and evaluation standards [50]. Thus, an improvement may be rather driven by a cut-point shift or psychological adaptation [51] reflecting the multitude of aspects that influence one’s subjective evaluation of their health. Han and Phillips [52] compared the predictive value of change in self-rated health with assessments of baseline self-rated health and ratings of self-rated health prior to death in community-dwelling disabled women aged 65 or older. A decline in self-rated health was significantly associated with increased mortality, while baseline self-rated health and self-rated health prior to death were not related controlling for relevant covariates. Also, in a cohort of Danes, a decline in self-rated health was associated with an increased mortality risk compared to unchanged good self-rated health. Notably, unchanged poor and improved self-rated health were also related to an increase mortality risk. The authors noted that poor self-rated health at any one measurement point was associated with an increased risk [49]. Change in self-rated health, specifically deterioration, were mainly predicted by low socio-economic status, poor quality of life, low trust in people and behavioural factors (e.g., moderate level of physical activity) or polypharmacy [53, 54].

However, to the best of our knowledge, the few prior studies analysing the association of change in self-rated health and mortality risk have not used data from Germany. Accordingly, there is a need for further research into the association between change in self-rated health and mortality risk among German older adults. The role of sex as a potential effect modifier of the relation of self-rated health and mortality has been neglected (e.g., 46). For instance, in our previous analysis we found that men rate their physical health better than women; the association of poor self-rated physical health to mortality was also stronger in men compared to women [34].

Thus, we aimed to extend prior findings by analysing levels of and change in self-rated health and their association with mortality over time in a German cohort and to determine the role of sex. Since previous research has shown, that self-rated physical health was most strongly associated with mortality [34, 36], we focused on self-rated physical health (SRPH).

Specifically, we:

1. Determined levels and change of SRPH in women and men of a large, aging community cohort.
2. Assessed whether and how change in SRPH affect mortality over baseline assessment while adjusting for major correlates of mortality (age, sex, socio-economic status, chronic physical diseases).
3. Test effect modification by sex on the relation between course of SRPH and mortality.

## Materials and methods

### Study design and participants

The present study used data of the Gutenberg Health Study (GHS), an ongoing population-based, prospective, observational single-center cohort study in the Rhine-Main region located in western Mid-Germany [55]. Primary endpoints are myocardial infarction and cardiovascular death. Overall mortality and diseases of the eye, the immune system, cancer, and mental health were defined as additional endpoints. The ethics committee of the Medical Chamber of Rhineland-Palatinate and the local and federal data safety commissioners approved the study protocol. The sample of the GHS was drawn randomly from the local registries of the city of Mainz and the district of Mainz-Bingen and stratified 1:1 for sex and residence and in equal strata across age decades. Further inclusion criteria were age 35 to 74, sufficient knowledge of the German language, and physical or mental ability to visit the study center for study investigations. Before inclusion in the study, written informed consent was obtained from each participant, according to the tenets of the Declaration of Helsinki. Inclusion criterion for the current investigation was participation at follow-up including filling out SRPH at baseline (T1, 2007–2012) and at 5-years follow-up (T2, 2012–2017) leading to a final analysis sample of *N* = 12,423 participants.

### Measures

To be able to analyze the effect of change in SRPH between T1 and T2 on mortality, all-cause mortality from T2 assessment onwards was defined as primary outcome. Updates of vital status were performed by quarterly queries to the registry offices and the mortality registry Rhineland-Palatinate. Reviews and death certificates with the exact date of death were acquired. For this analysis, mortality follow-up was complete as of December, 13, 2021, which results in a complete mortality follow-up for at least 5 five years in participants of T2 assessment with a median follow-up of 7.3 years due to administrative censoring only.

Self-rated physical was measured with the single-item “How would you rate your current physical health?” Response options ranged from very good to bad (1 = very good and 2 = good, 3 = not so good and 4 = bad). In line with widely used and valid single item questions assessing self-rated health [16, 17], the present single-item was previously formulated for the use in the GHS. Its validity has been demonstrated by relations to sociodemographic factors, chronic physical diseases and mortality in a current study. For instance, better SRPH was found among men and married participants. Moreover, while a positive association with SES was observed, negative associations with depression and anxiety symptoms as well as number of chronic physical diseases were determined [34]. We classified change of SRPH combining baseline and follow-up as one category by five classes: unchanged good/ very good, improved, unchanged not so good, deteriorated, and unchanged bad.

Chronic physical diseases were assessed in a computer-assisted personal interview at baseline and 5-year follow-up, cancer also after 7,5 years. Participants were asked whether they had ever (T1) received a definite diagnosis or whether they had received a definite diagnosis within the last five years (T2) of myocardial infarction, coronary artery disease, stroke, peripheral artery disease, atrial fibrillation, heart failure, cancer or chronic obstructive pulmonary disease by a physician. For the present analyses, numbers of chronic physical diseases reported at baseline and at follow-up were summarized to a sum score. Thirty participants were excluded from analysis due to incomplete SRPH. Disease information was incomplete in one item in 460 cases and on more than one item in 40 cases. For statistical analysis, the respective number of reported diseases we used as explanatory variable, irrespective of whether single items were incomplete. As the level of incompleteness was low, we did not apply more advanced imputation methods.

Sociodemographic characteristics were assessed as self-report and included age in years, sex as male and female, married [no/yes]. Combining data of education, profession and income, we defined socio-economic status (SES) according to Lampert, Kroll [56] ranging from 3 (lowest) to 21 (highest) SES.

### Statistical analysis

Descriptive characteristics were reported as absolute numbers and percentages for categorical variables and as means with standard deviations for continuous variables. Mortality was reported as proportions of deaths from T2 onwards. We fitted Cox proportional hazards models for assessing the predictive power of course of SRPH while adjusting for age, sex, SES and number of chronic physical diseases at T1 and T2. We further tested the interaction between sex and SRPH (ref. men with unchanged very good/good SRPH). The adequacy of entering age, SES and number of diseases as linear terms was checked by the method of fractional polynomials.

P-values were assumed to be significant with *p* < 001. Confidence intervals are reported at confidence level 95% without any adjustment for simultaneous error control. Statistical analyses were performed using SAS (2013) Statistical Analysis Software. Users’ Guide Statistics Version 9.4.

## Results

### Participants

A total of 12,423 participants filled out self-rated physical health at baseline and at follow-up. Their mean age was 54.5 years, and 49.8% were female. At T2, a total of 79.4% rated their physical health as very good/ good, while 2.5% reported bad SRPH. In women 76.8% indicated very good/good and 2.8% bad SRPH. 81.9% of men showed very good/good and 2.3% bad SRPH. During a median follow-up period of 7.3 (quartiles 6.0-8.5) years, 618 (5%) participants died.

Detailed information about participants’ sociodemographic and health characteristics, stratified according to mortality and sex, are shown in Table 1.

**Table 1:** Participants’ characteristics (stratified by mortality and sex)

*N*	Total	Alive	Died	*p*-value*	Females				Males			
					Total	Alive	Died	*p*-value*	Total	Alive	Died	*p*-value*
	12,423	11,805	618		6065	5879	186		6358	5926	432	
Variable												
Age (years, *M*,*SD*)	54.5 (10.9)	53.9 (10.7)	64.9 (8.0)	< 0.001	54.2 (10.8)	53.9 (10.7)	64.9 (8.0)	< 0.001	54.7 (10.9)	54.0 (10.7)	64.9 (8.0)	< 0.001
Sex (female, *N*, %)	6,065 (48.8)	5,879 (49.8)	186 (30.1)	< 0.001								
Married (*N*, %)	9,175 (74.0)	8,743 (74.2)	432 (70.2)	0.030	4240 (70.0)	4136 (70.5)	104 (56.2)	< 0.001	4935 (77.8)	4607 (77.9)	328 (76.3)	0.440
SES (*M*, *SD*)	13.2 (4.4)	13.3 (4.4)	11.8 (4.5)	< 0.001	12.5 (4.1)	12.5 (4.1)	10.6 (4.0)	< 0.001	14.0 (4.5)	14.1 (4.5)	12.3 (4.6)	< 0.001
Number of chronic physical diseases T1 (*N*, %)				< 0.001				< 0.001				< 0.001
*None*	9,856 (79.3)	9,495 (80.4)	361 (58.4)		4792 (79.0)	4687 (79.7)	105 (56.5)		5064 (79.7)	4808 (81.1)	256 (59.3)	
*1*	2,231 (18.0)	2,040 (17.3)	191 (30.9)		1120 (18.5)	1060 (18.0)	60 (32.3)		1111 (17.5)	980 (16.5)	131 (30.3)	
*2*	294 (2.4)	240 (2.0)	54 (8.7)		140 (2.3)	124 (2.1)	16 (8.6)		154 (2.4)	116 (2.0)	38 (8.8)	
*3*	40 (0.3)	28 (0.2)	12 (1.9)		13 (0.21)	8 (0.14)	5 (2.7)		27 (0.42)	20 (0.34)	7 (1.6)	
*>=4*	2 (0.0)	2 (0.0)	0 (0)		0	0	0		2 (0.03)	2 (0.03)	0 (0)	
Number of chronic physical T2 diseases (*N*, %)				< 0.001				< 0.001				< 0.001
*None*	10,815 (87.1)	10,448 (88.5)	367 (59.4)		5413 (89.3)	5292 (90.0)	121 (65.1)		5402 (85.0)	5156 (87.0)	246 (56.9)	
*1*	1,317 (10.6)	1,143 (9.7)	174 (28.2)		559 (9.2)	515 (8.8)	44 (23.7)		758 (11.9)	628 (10.6)	130 (30.1)	
*2*	236 (1.9)	179 (1.5)	57 (9.2)		77 (1.3)	60 (1.0)	17 (9.1)		159 (2.5)	119 (2.0)	40 (9.3)	
*3*	48 (0.4)	31 (0.3)	17 (2.8)		14 (0.23)	10 (0.17)	4 (2.2)		34 (0.53)	21 (0.35)	13 (3.0)	
*>=4*	7 (0.1)	4 (0.0)	3 (0.5)		2 (0.03)	2 (0.03)	0 (0)		5 (0.08)	2 (0.03)	3 (0.69)	
SRPH T1 (*N*,%)				< 0.001				0.022				< 0.001
*Very good/good*	10,172 (81.9)	9,708 (82.3)	464 (75.5)		4825 (79.6)	4686 (79.7)	139 (75.1)		5347 (84.1)	5022 (84.8)	325 (75.6)	
*Not so good*	1958 (15.8)	1,838 (15.6)	120 (19.5)		1069 (17.6)	1034 (17.6)	35 (17.6)		889 (14.0)	804 (13.6)	85 (19.8)	
*Bad*	285 (2.3)	254 (2.2)	31 (5.0)		168 (2.8)	157(2.7)	11 (6.0)		117 (1.8)	97 (1.6)	20 (4.7)	
*N.a.*	8	5	3		3	2	1		5	3	2	
SRPH T2 (*N*,%)				< 0.001				< 0.001				< 0.001
*Very good/good*	9,847 (79.4)	9,463 (80.3)	384 (62.3)		4653 (76.8)	4540 (77.3)	113 (60.8)		5194 (81.9)	4923 (83.2)	271 (63.0)	
*Not so good*	2,240 (18.1)	2,054 (17.4)	186 (30.2)		1233 (20.4)	1175 (20.0)	58 (31.2)		1007 (15.9)	879 (14.9)	128 (29.8)	
*Bad*	314 (2.5)	268 (2.3)	46 (7.5)		170 (2.8)	155 (2.6)	15 (8.1)		144 (2.3)	113 (1.9)	31 (7.2)	
*N.a.*	22	20	2		9	9	0		13	11	2	
Course of SRPH (*N*, %)				< 0.001				< 0.001				< 0.001
*Unchanged very good/good*	8,789 (70.9)	8,451 (71.7)	338 (55.1)		4096 (67.7)	3990 (68.0)	106 (57.3)		4693 (74.0)	4461 (75.5)	232 (54.2)	
*Improved*	1,192 (9.6)	1,131 (9.6)	61 (10.0)		791 (13.1)	751 (12.8)	40 (21.6)		693 (10.9)	591 (10.0)	102 (23.8)	
*Unchanged not so good*	854 (6.9)	793 (6.7)	61 (10.0)		637 (10.5)	626 (10.7)	11 (6.0)		555 (8.8)	505 (8.5)	50 (11.7)	
*Deteriorated*	1,484 (12.0)	1,342 (11.5)	142 (23.2)		483 (8.0)	460 (7.8)	23 (12.4)		371 (5.9)	333 (5.6)	38 (8.9)	
*Unchanged bad*	74 (0.6)	63 (0.5)	11 (1.7)		46 (0.8)	41 (0.7)	5 (2.7)		28 (0.4)	22 (0.4)	6 (1.4)	
*N.a.*	30	25	5		12	11	1		18	14	4	

Participant characteristics are shown as mean values and standard deviations or as percentages of available cases and absolute numbers. *M* = Mean; *SD* = Standard deviation; N.a.= not available; SES = Socioeconomic status; number of chronic physical diseases at T1 summarized myocardial infarction, stroke, peripheral artery disease, cancer, chronic obstructive pulmonary disease or heart failure until T1; number of chronic physical diseases at T2 summarized coronary artery disease, stroke, peripheral artery disease, cancer, atrial fibrillation, chronic obstructive pulmonary disease or heart failure over the last five years; SRPH = self-rated physical health; * by Satterthwaite t-test where mean and standard deviation is reported, chi-square-test elsewhere

### Change in SRPH

Overall, 70.9% of the participants indicated very good/good SRPH at T1 and T2, 6.9% reported not so good SRPH at T1 and T2, and 0.6% rated their SRPH as bad at both measurement points. 9.6% reported improvement and 12.0% deterioration of their SRPH. Among women, 67.7% reported unchanged very good/good SRPH, 10.5% indicated unchanged not so good SRPH, and 0.8 rated their SRPH as unchanged bad. An improvement of SRPH was reported by 13.1% of women, while 8.0% indicated a deterioration of their SRPH. 74.1% of men reported unchanged very good/good SRPH, 8.8% rated their SRPH at both measurements as not so good, and 0.4% indicated unchanged bad SRPH. An improvement of SRPH was found in 10.9% of men and deteriorated SRPH in 5.9%.

### Change in SRPH and mortality

In the total sample, among participants, who died, 55.1% had indicated an unchanged very good/ good SRPH, 10.0% reported not so good SRPH at both measurement points, and 1.7% indicated no change in their bad SRPH. While 10.0% of the participants reported an improvement of their SRPH, 23.2% of the participants, who died, indicated a deteriorated SRPH. In women, of those who died, 57.3% reported an unchanged very good/good SRPH, 6.0% indicated an unchanged not so good SRPH and in 2.7% was an unchanged bad SRPH found. While 21.6% the women, who died showed an improvement in their SRPH, 12.4% indicated a deterioration in SRPH. Among men, who died, 54.2% rated their SRPH at T1 and T2 as very good/good, 11.7% showed an unchanged not so good SRPH, and 1.4% reported bad SRPH at both measurements. While an improvement of SRPH was observed in 23.8% of men, who died, deteriorated SRPH was found in 8.9%.

Results of Cox proportional hazards models, showed that the course of SRPH was predictive for mortality risk after adjusting for age, sex, SES, and known physical diseases (Table 2). SRPH at T1 showed predictive only without including SRPH at T2 (*p* = 0.006). As judged by likelihood ratio testing, adding baseline SRPH did not improve model fit when added to the SRPH at T2 (*p* = .029). There was a significant interaction between sex and SRPH (Likelihood ratio chi square *p* = 0.022 comparing the model with vs. without interaction). Overall, hazard ratios of men were mostly in the same direction but higher compared to women. The highest hazard ratio for mortality were found for unchanged bad (men: HR: 2.28; 95% CI: 1.00 to 5.19%; women: HR: 2.01; 95% CI: 0.82–4.95); deterioration (men: HR: 1.87; 95% CI: 1.47 to 2.38; women: HR: 1.37; 95% CI: 0.95 to 1.98), followed by unchanged not so good (men: HR: 1.47; 95% CI: 1.04 to 2.08; women: HR:1.33; 95% CI: 0.84 to 2.09). For improvement we found divergent effects: in men, improvements were associated with a 1.67-fold increase in mortality (HR: 1.67; 95% CI: 1.22 to 2.27), while women, who reported an improved SRPH, had a lower mortality risk (HR: 0.57%; 95% CI: 0.31 to 1.06) compared to unchanged very good/good SRPH in men and women, respectively. For detailed information see Table 2. Note, that further differentiating between very good and good SRPH did not improve model fit as judged by likelihood ratio test.

**Table 2:** Results of cox proportional hazards models for assessing the common effect course of SRPH on mortality while adjusting for age, sex, SES, number of chronic physical diseases and cancer, and for effect modification by sex

Variables	Model 1 Common effect of course of SRPH		Model 2 Effect of course of SPRH modified by sex			
	*HR (95% CI)*	*p*-value*	*HR (95% CI)*		*p*-value*	
Age	1.10 (1.08–1.11)	< 0.001	1.10 (1.08–1.11)		< 0.001	
Sex (Ref.= male)	0.45 (0.38–0.54)	< 0.001	0.54 (0.43–0.68)		< 0.001	
SES	0.97 (0.95–0.99)	< 0.001	0.97 (0.95–0.99)		< 0.001	
Number of chronic physical diseases (T1)	1.24 (1.09–1.40)	0.001	1.23 (1.09–1.40)		0.001	
Number of chronic physical diseases (T2)	1.53 (1.39–1.69)	< 0.001	1.53 (1.38–1.69)		< 0.001	
Cancer	1.26 (1.04–1.53)	0.016	1.28 (1.06–1.54)		0.012	

**Course of SRPH**	*HR (95% CI)*	*p*-value*	Females		Males	
			*HR (95% CI)*	*p*-value*	*HR (95% CI)*	*p*-value*
		<0.001		0.037		< 0.001
*Unchanged very good/good (Ref.)*	1		1		1	
*Improved*	1.25 (0.95–1.65)		0.57 (0.31–1.06)		1.67 (1.22–2.27)	
*Unchanged not so good*	1.43 (1.08–1.89)		1.33 (0.84–2.09)		1.47 (1.04–2.08)	
*Deteriorated*	1.71 (1.39–2.09)		1.37 (0.95–1.98)		1.87 (1.47–2.38)	
*Unchanged bad*	2.21 (1.20–4.07)		2.01 (0.82–4.95)		2.28 (1.00-5.19)	

*N* = 12,342; events = 611; HR = Hazard ratio; 95% CI = 95% confidence interval; *SD* = Standard deviation; SES = Socioeconomic status; SRPH = Self-rated physical health; number of chronic physical diseases at T1 summarized myocardial infarction, stroke, peripheral artery disease, cancer, chronic obstructive pulmonary disease or heart failure until T1; number of chronic physical diseases at T2 summarized coronary artery disease, stroke, peripheral artery disease, cancer, atrial fibrillation, chronic obstructive pulmonary disease or heart failure over the last five years; the cancer-variable summarized information of a diagnosis at T1, T2 and information of a computer-assisted personal interview 2,5 years after T2; effect modification by sex was assessed relative to men with unchanged very good/good SRPH; * by Wald chi-square test, Likelihood ratio chi-square test for interaction of SRPH by sex *p* = 0.022

## Discussion

Since the 1950s, self-rated health has been recognized as an important global health indicator in predicting future health-related events and use of medical care, especially the dimension of self-rated physical health [15, 34, 36]. However, most findings relied on baseline measures of self-rated health. The role of sex in this association has hardly been explored. Thus, the mid- and long-term predictive power of self-rated health and its change over time is still unclear. The present work aimed to assess the course of SRPH in an aging population over time and associate it with mortality in men and in women. While the number of chronic physical diseases increases when getting older, we found a remarkable average stability of very good/good self-rated physical health over five years in the great majority of 70.9% of participants. Nevertheless, 0.6% reported bad SRPH at both measurement points and a sizeable subgroup of 12.0% indicated a deteriorated SRPH. Sex-stratified analyses mirrored similar patterns: Most women and men rated their SRPH at both measurements as very good/good (67.7%; 74.0%). While 0.8% of women and 0.4% of men reported unchanged bad SRPH, deterioration was showed by 8.0% of women and 5.9% of men. Notably, in the total sample, participants with an unchanged bad SRPH or a deterioration of SRPH were at highest risk of mortality compared to participants indicating very good/good SRPH at both measurement points, even after taking relevant confounders into account. Moreover, additional analyses demonstrated, that SRPH at T1 was predictive only without including SRPH at T2. Further, adding SRPH at T1 to SRPH at T2. Similar patterns were found in the sex-stratified analyses. As in a previous, cross-sectional paper, associations were stronger for men than for women. However, specifying previous findings on the effect modification by sex [45, 49], results of interactions in a Cox proportional hazards models showed that, an improvement in SRPH increased the risk compared to an unchanged very good/ good SRPH, but only in men. In women we found the reverse association, declined mortality in the case of improved SRPH. These findings indicate that men and women may rate their health differently. As we had previously shown [34], men rated their physical health better, but suffered from a higher mortality than women. Possibly, unlike women, they also may overrate their physical health after having endured and mastered a significant health crisis [46]. Further, findings may also reflect a stronger response shift in males [50]. Higher age, male sex, lower socio-economic status and chronic physical disease at T2 were predictive of mortality. Overall, our findings are in line with previous findings showing that poor self-rated health ratings were associated with higher mortality compared to participants with good self-rated health, even when controlling for relevant confounders (e.g., 27, 28, 29, 34). Further, results of the present work supported the assumption of [15], that self-rated health capture health aspects relevant to mortality which are not covered by clinical assessment. Consistent with findings on change of self-rated health, we found an improved prediction of mortality when repeated assessments of SRPH were taken into account. Comparable to Beth et al. (2005), our findings suggested that, a decline in SRPH was significantly associated with increased mortality risk, even when controlling for relevant confounders.

### Strength and limitations

Strengths of the present study referring to its longitudinal study design with repeated measurements from a large-scale cohort study. Further, the use of one specific component of self-rated health enabled us to analyse the specific predictive value of the physical aspect. Thus, findings may target preventive health policies as well as to clarify understanding of the overall construct of self-rated health. However, the findings must be interpreted considering the study’s limitations. About one third of the regional population were willing to participate which resulted in some selection toward higher social status, less migrant background and possibly with respect to health status at study entry. As in other large cohorts no selection with respect to outcomes was effective and a bias in exposure risk relations would be plausible only in presence of strong interaction effects. Neither sex nor SES showed such behaviour. As data of the present study were gathered within the German general population, the generalizability to other countries is still unclear. Results may also change when different operationalization were used to capture change in SRPH. For instance, instead of using computed change, change can also be operationalized as retrospectively reported change by asking participants if they feel that their health has improved, declined, or stayed constant between two measurement points [46]. We used all-cause mortality as outcome, therefore we were not able to assume prediction on cause-specific mortality. Further, our results suggested that even an improvement in SRPH increased the risk compared to an unchanged very good/ good SRPH. However, we have to concede that we did not take differences of distinct courses of improvement into account. For instance, an improvement from bad to not so good may had different effects on mortality compared to an improvement from not so good to very good/good. To aim at a better understanding of underlying mechanisms, future research should integrate cause-specific mortality data into the analysis and differentiate courses of improvements. Nevertheless, improvements should be interpreted critically taking further evaluating aspects into account. It should be kept in mind, that mortality risk is higher even if precious health conditions may normalize. Further, we concede that older adults who lived in, or moved to nursing homes, respectively, and those who were house-bound were presumably underrepresented as study participation required on-site visits. Also, our set of correlates may not be sufficient. For instance, some researchers pointed to a difference of self-rated health among rural and urban residents [57, 58]. The present study did not take this into account. Our study was based on a proposal that contained a statistical analysis plan, including a limited list of covariables to be considered. Physical disease, sex and SES were considered the most relevant variables. We had learned from similar investigations on the health status and SRPH at baseline, that the number of serious chronic conditions as documented in the GHS captures essential prognostic information. Thus, we performed a number of sensitivity analyses to make sure that no variation of disease variable coding provided more information. We did so by comparing refined models with a basic model via likelihood ratio testing. Further, the interplay between physical and mental health with anxiety and depression could be considered in future studies.

## Conclusion

Finding of the present work pointed to potential adjustment to chronic physical diseases. Indeed, maintaining self-rated physical health in the face of diseases may indicate healthy ageing. However, a sizeable subgroup reported deterioration of SRPH. Data of the present work extend previous findings showing that low levels of SRPH, especially a deterioration, is associated with increased mortality in the long run. Thus, it can be assumed, that previous well-established associations between SRPH and mortality might have been underestimated mortality risk for individuals with recent change in SRPH. They endorse the view that SRPH is an important additional health indicator beyond clinical assessment which should be checked in medical consultations, particularly in elderly patients routinely. It can be included as a simple question in doctor visits or surveys. Further, change in SRPH can be easily created with longitudinal data.

## Data Availability

Written informed consent from GHS study participants does not allow publicaccess to the data. Access to the data in the local database is possible at anytime upon request according to the ethics vote. This concept was developed withthe local data protection officer and the ethics committee (local ethicscommittee of the Rhineland-Palatinate Medical Association, Germany).Interested scientists can make their requests to the Gutenberg Health StudySteering Committee (e-mail: info@ghs-mainz.de).

## Abbreviations

SRPH: Self-rated physical health
GHS: Gutenberg Health Study
SES: Socio-economic status
